# PsyCoP – A Platform for Systematic Semi-Automated Behavioral and Cognitive Profiling Reveals Gene and Environment Dependent Impairments of Tcf4 Transgenic Mice Subjected to Social Defeat

**DOI:** 10.3389/fnbeh.2020.618180

**Published:** 2021-01-14

**Authors:** Paul Volkmann, Marius Stephan, Sven Krackow, Niels Jensen, Moritz J. Rossner

**Affiliations:** ^1^Department of Psychiatry and Psychotherapy, Laboratory of Molecular Neurobiology, University Hospital, Ludwig-Maximilians-University Munich, Munich, Germany; ^2^International Max Planck Research School for Translational Psychiatry (IMPRS-TP), Munich, Germany; ^3^Institute of Pathology and Molecular Pathology, University Hospital Zurich, Zurich, Switzerland

**Keywords:** psychiatry, gene × environment interaction, psychosocial stress, intellectual disabilities, schizophrenia, research domain criteria

## Abstract

Recently, hundreds of risk genes associated with psychiatric disorders have been identified. These are thought to interact with environmental stress factors in precipitating pathological behaviors. However, the individual phenotypes resulting from specific genotype by environment (G×E) interactions remain to be determined. Toward a more systematic approach, we developed a novel standardized and partially automatized platform for systematic behavioral and cognitive profiling (PsyCoP). Here, we assessed the behavioral and cognitive disturbances in *Tcf4* transgenic mice (*Tcf4*tg) exposed to psychosocial stress by social defeat during adolescence using a “two-hit” G×E mouse model. Notably, *TCF4* has been repeatedly identified as a candidate risk gene for different psychiatric diseases and *Tcf4*tg mice display behavioral endophenotypes such as fear memory impairment and hyperactivity. We use the Research Domain Criteria (RDoC) concept as framework to categorize phenotyping results in a translational approach. We propose two methods of dimension reduction, clustering, and visualization of behavioral phenotypes to retain statistical power and clarity of the overview. Taken together, our results reveal that sensorimotor gating is disturbed by *Tcf4* overexpression whereas both negative and positive valence systems are primarily influenced by psychosocial stress. Moreover, we confirm previous reports showing that deficits in the cognitive domain are largely dependent on the interaction between *Tcf4* and psychosocial stress. We recommend that the standardized analysis and visualization strategies described here should be applied to other two-hit mouse models of psychiatric diseases and anticipate that this will help directing future preclinical treatment trials.

## Introduction

Most psychiatric diseases such as autism spectrum disorder, major depression, and schizophrenia are thought to arise from complex interactions of genetic and environmental influences (Lai et al., 2014; Weinberger, 2017; Calabrò et al., 2019). Pre- and perinatal infections as well as psychosocial stress during childhood and adolescence are important environmental risk factors (Brown et al., 1999; Allswede and Cannon, 2018; Assary et al., 2018; Richetto and Meyer, 2020). Despite different clinical symptoms, psychiatric diseases share similar genetic factors and psychopathological characteristics (Calabrò et al., 2020). However, not much is known about the complex interactions of all these risk factors in the pathogenesis of psychiatric symptoms and diseases. While patient-derived induced pluripotent stem cells (iPSCs) are used to study the influence of a patient-specific set of multiple genetic factors at the cellular level, the impact of genotype by environment (G×E) interactions on behavior can only be studied in animals (Tsuang, 2000; van Os et al., 2010; Hosák and Hosakova, 2015).

In the past, advancing results from animal models into the clinic have frequently failed (Markou et al., 2009; Nestler and Hyman, 2010; Jones et al., 2011), emphasizing the need to improve the validity of disease models. For major depression and schizophrenia, it has been postulated that two hits, i.e., the combination of a genetic predisposition and an environmental impact, are necessary for developing these disorders (Maynard et al., 2001; Tost and Meyer-Lindenberg, 2012). Both diseases have a strong genetic background, but concordance rates in monozygotic twins are around 50–80% that indicates substantial environmental contributions as well.

The first hit, i.e., the genetic predisposition, has recently been addressed by several genome-wide association studies (GWAS), copy number variation analyses, as well as exome sequencing approaches and hundreds of risk gene loci with variable effect sizes have been identified (Marshall et al., 2017; Pardiñas et al., 2018; Li et al., 2020). Among the best, repeatedly found, “cross-disorder” pleiotropic risk genes for neurodevelopmental psychiatric disorders is *Transcription Factor 4* (*TCF4*) that has been shown to be implicated in autism spectrum disorder, major depression, and schizophrenia (Forrest et al., 2018; Pardiñas et al., 2018; Amare et al., 2019; Calabrò et al., 2020). The gene product of *TCF4* is a basic helix-loop-helix (bHLH) transcription factor involved in developmental and plasticity-related transcriptional programs in the CNS, including pathways essential for cognition and learning (Li et al., 2019; reviewed in detail in Quednow et al., 2014). TCF4 mediates cell proliferation and neurite growth-associated processes, influences excitability in manifold ways (D'Rozario et al., 2016; Rannals et al., 2016; Page et al., 2018), and acts as a transcriptional hub for several bHLH proteins (Quednow et al., 2014). TCF4 binding sides have been identified across a large repertoire of genes including many risk factors for schizophrenia and other neurodevelopmental disorders (Forrest et al., 2018). *TCF4* is located on chromosome 18 in mice and humans. Haploinsufficiency leads to severe intellectual disability and retardation in the Pitt-Hopkins-Syndrome type of autism spectrum disorder (Goodspeed et al., 2018) and more 5′ located risk alleles associated with long *TCF4* isoforms influence performance in certain cognitive tasks (Albanna et al., 2014). *Tcf4* knock-out mice suffer from severe brain defects (Flora et al., 2007; Li et al., 2019), whereas moderately modulating the expression of long *Tcf4* isoforms in gain and loss of function mouse models is known to cause cognitive impairments (Brzózka et al., 2010; Brzózka and Rossner, 2013; Quednow et al., 2014; Badowska et al., 2020).

The second hit are environmental conditions – non-genetic factors during vulnerable developmental phases (Schneider, 2013). The identification of such environmental influences is difficult, but several conditions have been associated with an increased risk of developing schizophrenia, including migration, urban upbringing, and childhood trauma, pointing at the contribution of psychosocial stress (Holz et al., 2020).

Cognitive impairments in *Tcf4* gain and loss of function mouse models were indeed shown to be worsened by chronic psychosocial stress or isolation rearing, making this a promising two-hit mouse model (Brzózka et al., 2010; Badowska et al., 2020).

Another strategy to improve the validity of genetic animal models, particularly for genes that affect several diseases such as *Tcf4*, is to focus on neurophysiological endophenotypes instead of clinical symptoms. The Research Domain Criteria (RDoC) system offers a valuable classification framework for this (Insel et al., 2010) that consists of the following domains: cognition, social processes, sensorimotor systems, positive and negative valence, as well as arousal and regulatory systems (Morris and Cuthbert, 2012). These domains match brain phylogeny and are based on distinct biological brain systems and networks (Anderzhanova et al., 2017). The RDoC framework was specifically developed for improving the translational value of neurobiological and psychiatric animal models. However, no systematic evaluation in this context has been attempted so far. We are presenting here a comprehensive behavioral profiling in accordance with the RDoC concept using existing mouse models called PsyCoP (platform for systematic behavioral and cognitive profiling).

We have used PsyCoP to profile *Tcf4* transgenic mice (*Tcf4*tg) subjected to social defeat at adolescence as a “two-hit” G×E mouse model. The test battery consisted of a diverse panel of well-established behavioral tests. For the R script based automated analysis according to the behavioral traits and domains defined in RDoC framework we have used family-wise group comparisons as well as dimension-reduction visualizations analyses. Using this workflow, we could demonstrate a strong synergistic contribution of psychosocial stress specifically on the development of cognitive dysfunction and novelty-induced hyperactivity in the *Tcf4* overexpression mouse model. Whereas, sensorimotor gating was impaired by the genetic factor only and the positive and negative valence systems were solely affected by psychosocial stress. Moreover, we present a generalizable approach for improving the predictive validity and translatability of psychiatric mouse models.

## Materials and Methods

### Animals and Husbandry

C57Bl/6N mice were obtained from Charles River Laboratories GmbH, Sulzfeld, Germany. The *Tcf4* transgenic and wildtype mice were kept on a C57Bl/6N background.

Mice were kept under a 12:12 light/dark cycle matching the natural day/night cycle. Lights were switched on at 7 a.m. throughout the testing period. Food was provided *ad libitum*; water was conditionally restricted in the IntelliCage System only. Male *Tcf4* transgenic (*Tcf4*tg) and wildtype (wt) littermate control mice were weaned at 21 days and group housing was established in groups of ~10 male animals of mixed genotypes. Separate groups were used for psychosocial stress and no stress control. Animals were kept in type IV cages (Tecniplast 2000, 612 × 435 × 216 mm, 2,065 cm^2^). Test mice were 9–14 weeks of age, when used for subsequent experiments.

### Experimental Procedures

**Figure 1:**
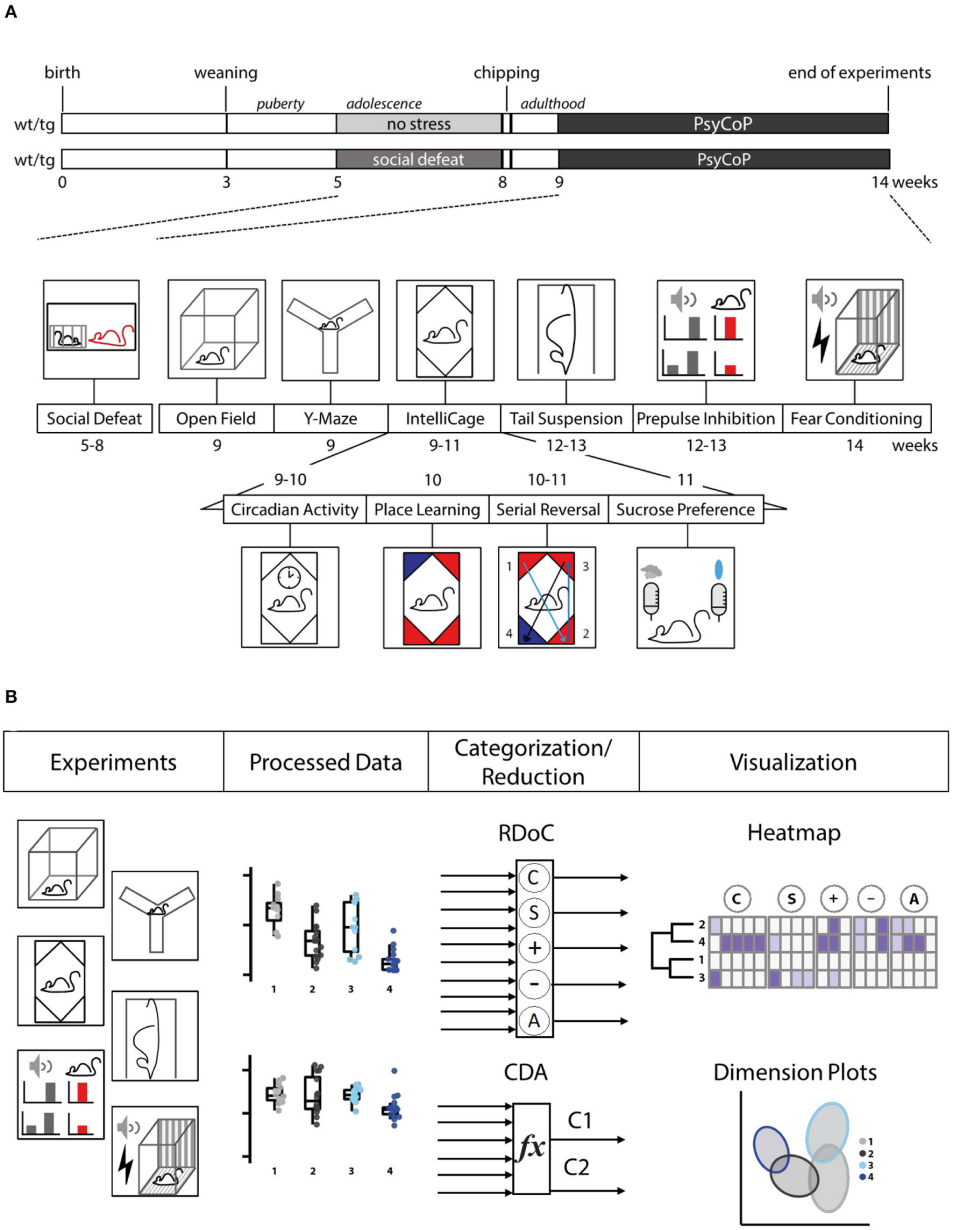
Study design. **(A)** Timelines for *Tcf4*tg (tg) and wildtype (wt) mice subjected to social stress during adolescence (social defeat; sd) or not (no stress control, ns). Behavioral testing took place during weeks 9–14, then RFID chips were implanted, and the animals subjected to behavioral tests in order of increasing aversiveness. **(B)** Schematic overview of the analysis pipeline. After semi-automated data processing, single variables were visualized and overall neurocognitive profiles were generated by categorization in accordance with the RDoC framework, resulting in a heatmap and dimension reduction *via* canonical discriminant analysis (CDA), resulting in a dimension plot.

Experimental mice were moved with clear polycarbonate tunnels to avoid the stress induced by classical tail handling (Hurst and West, 2010). Mouse studies were conducted in accordance with the German Animal Protection Law. Animals were habituated in the experiment room for at least 10 min. All experiments were conducted during the light phase, except IntelliCage experiments, which ran continuously. Test equipment was cleaned using SDS solution and then ethanol before and after usage if not stated otherwise. Animals were exposed to the following tests consecutively (Figure 1).

### Social Defeat

The resident intruder social defeat paradigm of psychosocial stress was essentially performed as described in Brzózka et al. (2011), starting at an age of 34–42 postnatal days. FVB/N male mice, 45–70-week-old, were used as resident stressor mice. Male residents were primed by pairing with a female of the same age for 3 days and their attack latency was assessed with 8 weeks old C57Bl/6N wildtype male mice. Only residents with attack latencies under 20 s were used. On 21 consecutive days, starting at 5 weeks, experimental mice (intruders) were taken out of their home cage individually and inserted into a resident's home cage for a total of 21 sessions for each intruder test mouse. After the first physical attack, animals were protected by a perforated metal cage (75 mm × 115 mm × 60 mm) for another 60 min. None of the animals suffered bite marks or showed other signs of injury. The intruders were then identified by their ear tags, their tails marked with a waterproof pen, and put back into their home cage. Metal cages were cleaned with water and ethanol between sessions. The test time was randomized daily between 7 a.m. and 7 p.m. The pairing residents to intruders was rotated in order to avoid repeated contacts more than once within 12 days and more than twice total. The chronological order of intruder cages was changed daily. Mice from the no stress control groups were taken out of the cages and handled 3 times daily for the last 3 days of the social defeat period for habituation. As social defeat served the purpose of exposing animals to psychosocial stress, no data was collected, and animals were only observed by the experimenter.

### Transponder Implantation

Test animals were identified by implanted RFID transponders using a handheld scanner and within the IntelliCage. The transponders were implanted after the psychosocial stress period and 1 week before starting IntelliCage experiments to ensure complete recovery from surgery. Mice were anesthetized using isoflurane and then shaved in a small area in the dorsocervical region. Eyes were covered with Dexpanthenol eye ointment, the skin was disinfected with 70% alcohol, the transponder (1.4 × 9 mm) was placed subcutaneously in the neck region, and the wound was closed with one to two stitches.

### Open Field Test

Open field test was essentially performed as described previously (Hühne et al., 2020) except that testing was started at zeitgeber time (ZT) 2, 2 h after lights turned on. A short description of the standard operating protocol can be found in Supplementary Material.

### Y-Maze Test

The Y-maze-Test was performed as described in Hühne et al. (2020). A short description of the standard operating protocol can be found in Supplementary Material.

### IntelliCage System

The IntelliCage system (http://www.tse-systems.com/product-details/intellicage) consists of a frame put into type 4 cages as described above. The frame is composed of four corners with a door on each of two sides. When opened, mice could access a water bottle behind these doors. In case doors were closed, mice could open the doors (depending on the current paradigm) by poking at a given door, disrupting a light barrier. Only one mouse was usually present in a corner, at a time. Visits to a corner, nosepokes and licks at water bottle nipples were monitored continuously throughout all IntelliCage experiments.

Mice were introduced to the IntelliCage on the day of Y-maze-testing at ZT9. Cage assignments were maintained after transfer to the IntelliCage, as animals remained in the established groups.

The order of experimental phases was as follows (1 day referring to a timespan of 24 h, experiments were switched during the light phase):

▪ 2 days of Free Adaptation: open doors and free access to water bottles in all corners;
▪ 1 day of Free Adaptation, doors open on visit: doors open when a mouse entered a corner, free access to water bottles in all corners;
▪ 2 days of Nosepoke Adaptation: doors stayed closed until a mouse entered the corner and executed one or more nosepokes on a door; only the corresponding door opened and granted access to water for 7 s or until the mouse withdrew from the corner;
▪ 1 day of Nosepoke Adaptation at 50%: same procedure as for Nosepoke Adaptation, but doors only opened with a probability of 50% for each trial to counteract a bias for a specific corner by making it unreliable;
▪ 1 day of Nosepoke Adaptation at 30%: same procedure as for nosepoke adaptation, but doors only opened with a probability of 30% for each trial;
▪ 2 days place learning: each mouse was assigned to one of the four corners in a balanced fashion, ensuring equal distribution; mice could only get access to water after executing a nosepoke in their assigned corner;
▪ 7 days serial reversal learning: place learning with a new assignment of the drinking corner for each mouse every 24 hours, following a pre-defined order to make the new corner unpredictable for the mouse;
▪ 1 day sucrose preference: doors were open with free access to water bottles in all corners; one of the two bottles in each corner (each on the same side) was filled with a 4% sucrose solution, the other bottle contained normal drinking water.

Place and sucrose preference as well as nocturnality of general activity were measured using the preference score (A-B)/(A+B), weighted for random expectation, where A equals the number of correct trials (visits in the assigned corner with at least one nosepoke), the number of licks at a sucrose solution bottle or visits during nighttime and B equaling incorrect trials (visits with nosepoke in non-assigned corner), licks at a bottle containing plain water or daytime visits.

Sequential Probability Ratio Testing (SPRT) was used to calculate the learning criterion for the assessment of learning performance over the serial reversal learning phases and learning flexibility after the first reversal (Wald, 1945). The learning criterion is the number of trials—defined as visits with at least one nosepoke—needed to pass the upper bound, i.e., a predefined learning criterion. The SPRT upper bound was defined as random expectation plus 10% (35% for four corners), the lower bound was equal to random expectation (25% for four corners). Significance levels were set to 5% for both bounds. In case a mouse did not reach the learning criterion within the duration of a reversal phase (24 h), the total number of trials was used for downstream analysis and plotting instead. Overall, serial reversal learning performance was measured as the approximated area under the curve across all reversal phases.

### Pre-pulse Inhibition

Pre-pulse inhibition was essentially performed as described in Hühne et al. (2020). Startle responses were assessed using software from SR-LAB (San Diego Instruments, San Diego, USA). In advance to the actual testing, mice were habituated to the test boxes for 10 min on three consecutive days. Testing was performed on day 4. A short description of the standard operating protocol can be found in Supplementary Material.

### Tail Suspension Test

The tail suspension test was performed as described in Hühne et al. (2020). A short description of the standard operating protocol can be found in Supplementary Material.

### Fear Conditioning

Fear conditioning was performed as described in Brzózka et al. (2016). A short description of the standard operating protocol can be found in Supplementary Material.

### Statistical Analysis

In all experiments except for social defeat, IntelliCage experiments, and pre-pulse inhibition, mice were video-recorded and behavioral measures were quantified using the tracking software ANY-maze (Stoelting, Wood Dale, IL, USA). Raw data from different experimental software was analyzed using the R based, automated user interface FlowR (XBehavior, Dägerlen, Switzerland). Statistical tests were calculated, and graphs were plotted using RStudio (RStudio, Boston, MA, USA).

Eight animals had to be taken out of the experiment prematurely because of biting injuries. Due to technical errors, two obviously incorrect measurements had to be excluded from analysis.

Boxplots were created using the R function *ggplot* from the package *ggplot2* (Wickham, 2016). Whiskers extend no more than 1.5 times the interquartile range (IQR) from their hinges. Datapoints with a distance of <3% of the total range were shifted horizontally by a random offset. *P*-values refer to two-way analyses of variance (ANOVA) with Type 2 sum of squares.

Briefly, a multivariate bifactorial linear model was fitted to the untransformed complete dataset and tested for normality with the E-test for multivariate normality implemented as parametric bootstrap in the *energy* R-package with 1000 bootstrap replicates as proposed in Székely and Rizzo (2005). Additionally, a QQ plot and a density plot of the scaled residuals can be found in Supplementary Figure 3. This model was tested in a multivariate analysis of variance (MANOVA) with *F*-values from a Wilk's lambda approximation. From the same, statistic univariate comparisons were derived, and the resulting *p*-values were adjusted for multiple comparisons by false discovery rate (FDR) adjustment. In case of a significant interaction term, the corresponding one-way ANOVA was calculated and indicated for each genotype level. This tests for an environment effect without any genotype influence. Detailed test results can be found in Supplementary Table 3.

For repeated measures two-way ANOVAs, a linear model was constructed and its residuals were tested for normality with an E-test with 1000 bootstrap replicates (Székely and Rizzo, 2005). F-values were calculated with Wilk's lambda approximation. Homogeneity was tested for the respective within group variable with Mauchly's sphericity test and *p*-values were corrected using the Huynh-Feldt procedure in case of a significant violation. After finding a significant interaction of the genetic and environmental factor, we conducted subsequent repeated measures one-way ANOVAs for each genotype level.

Heatmaps were created using the R function *pheatmap* from the *pheatmap* package. Data in **Figure 5** was *z*-transformed for clustering. The applied clustering method for the groups used the complete linkage for hierarchical clustering of Manhattan distances (Strauss and von Maltitz, 2017), defining the distance of two clusters as the maximum distance between their individual components (Ward, 1963).

The color code refers to the distances of group mean *z*-scores to wt no stress control as reference. Column blocks were defined by RDoC domains (Anderzhanova et al., 2017). Variables were assigned to RDoC domains *a priori* based on the reference matrix provided by the National Institute of Mental Health (NIMH RDoC Matrix).

For the Principal Component Analysis (PCA) (Supplementary Figure 2), the R function *nipals* from the *ade4* package was used. *nipals* executes a non-linear iterative partial least squares (NIPALS) algorithm for dimension deflation and imputation of missing values. It was used not only for PCA, but also to gain a reconstituted dataset with estimators for missing values, which was subsequently normalized by *z*-transformation for downstream analysis. For plotting, the R function *ggplot* from package *ggplot2* (part of *tidyverse*) was used. Similar to CDA, two plots were generated for the first two principal components (PCs): one with single data points for Principal Components 1 and 2 as well as data ellipses; and one without individual data points, but the same ellipsoids for PCs 1 and 2 as well as vectors indicating the contribution of single variables (experiments) to each principal component.

For Canonical Discriminant Analysis (CDA), the R function *candisc* from the *candisc* R package was used and results were plotted with *ggplot2*. The *z*-transformed NIPALS-derived data matrix from the PCA workflow was used as input. Briefly, in a CDA, linear combinations (canonical components) of variables are calculated, providing maximal separation of groups. This is done in four steps: The pooled within-group covariance matrix is converted into an identity matrix; the group means of transformed variables are calculated; a Singular Value Decomposition (SVD) of the means is calculated, weighting for the sample size of each group; finally, the resulting eigenvalues are back-transformed to the original variable space, yielding canonical components. These components summarize variation between groups similar to the way PCA summarizes overall variation. The corresponding plots display the first two canonical components with, again, individual data points and data ellipses in the first, and with the same ellipsoids and vector representations of canonical coefficients in the second plot.

A heatmap of the canonical coefficients of the CDA for each term of the multivariate linear model was created with *pheatmap*.

## Results

In this study, we have developed a neurocognitive test battery and analysis workflow called PsyCoP and applied it to investigate the effects of a combination of genetic disposition and environmental impact in a psychiatric mouse model. The study was designed as a two by two (2 × 2) factorial experiment with wildtype animals kept as no stress controls during their adolescence representing the healthy control group (wt ns) and *Tcf4* gain of function mice exposed to psychosocial stress as a putative disease model (tg sd; Figure 1A). Stressed wildtype mice (wt sd) as well as non-stressed *Tcf4*tg mice (tg ns) served as controls for the contribution of each of these factors alone. The behavioral tests were arranged in order of increasing aversiveness (Figure 1A). The whole dataset was analyzed by categorization into the corresponding RDoC domains and into neurocognitive profiles after dimension reduction (Figure 1B).

### Cognitive Deficits of *Tcf4*tg Mice Are Increased by Psychosocial Stress

#### Cognitive Systems

**Figure 2:**
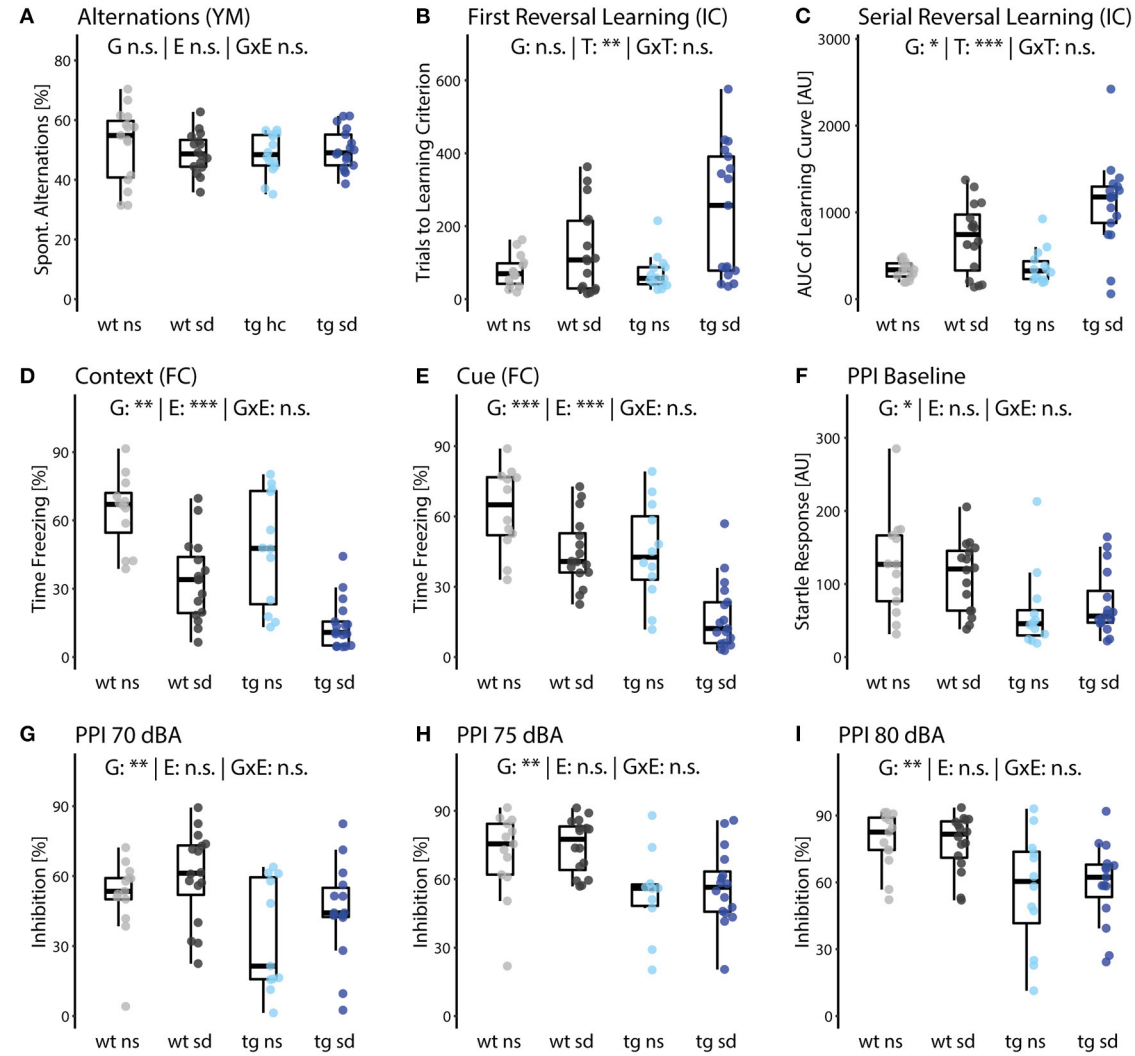
Cognitive deficits but not sensorimotor gating impairments of *Tcf4*tg mice are increased by psychosocial stress. **(A)** The Y-maze test showed no significant differences in spontaneous alternation between groups. **(B,C)** First [**B**; E: *F*_(1,49)_ = 11.0, *p* = 0.0065] and serial reversal [**C**; E: *F*_(1,49)_ = 29.8, *p* = 4.47E-5] measures were lower for the social defeat (sd) than for the no stress control (ns) groups, while the genotype effect was only significant in serial reversal [G: *F*_(1,49)_ = 7.18, *p* = 0.034]. **(D,E)** Fear conditioning (FC) revealed a highly significant reduction in contextual [E: *F*_(1,49)_ = 38.26, *p* = 6.91E-6] and cued [E: *F*_(1,49)_ = 22.3, *p* = 2.8E-4] memory measures in sd, which is additive to a similar reduction in *Tcf4*tg mice (tg) compared to wildtype (wt) mice [G: **(D)** *F*_(1,49)_ = 13.0, *p* = 0.0043; **(E)** *F*_(1,49)_ = 24.1, *p* = 2.8.E-5]. **(F–I)** Pre-pulse inhibition (PPI) measures were not affected by treatment but differed highly significantly between genotypes [G: **(F)** *F*_(1,49)_ = 8.37, *p* = 0.023; **(G)** *F*_(1,49)_ = 11.6, *p* = 0.0058; **(H)** *F*_(1,49)_ = 13.9, *p* = 0.0040; **(I)** *F*_(1,49)_ = 15.0, *p* = 0.0031]. Data are shown as box plots with whiskers extending to no more than 1.5-fold IQR; *p < 0.05, **p < 0.01, ***p < 0.001, n.s., not significant; *p*-values are FDR-corrected and refer to Wilk's lambda testing two-way ANOVA; *n* = 15/17/15/17. G, genotype term; E, environment term; G×E, interaction term; FC, fear conditioning; PPI, pre-pulse inhibition.

To assess the working memory capacity of the mice, we applied the Y-maze test. The test exposes animals to simple and unforced choices between different arms they can enter within a Y-shaped maze. Spontaneous alternations describe the animal's success in choosing the arm they have not visited recently. We did not identify any differences between groups (Figure 2A), showing that neither *Tcf4* gain of function nor social defeat influenced this trait.

However, learning tasks performed in the IntelliCage revealed differences between the four groups: after 48 h in a place learning setup, in which animals were only granted access to water in an individually assigned corner, we altered assignments of individual corners for each animal. Learning flexibility was assessed by measuring the number of trials needed to reach a pre-defined learning criterion after a first reversal of the correct corner, whereas the dynamics of the learning progress were measured as the area under the learning curve over all six reversal phases. In both parameters a lower value indicates higher learning speed and thus better learning performance. Additionally, a pseudo-time course of the serial reversal learning experiment can be found in Supplementary Figure 1. While we did not observe significant differences between wildtypes and *Tcf4* transgenic mice after the first reversal, social defeat had a clear impact (Figure 2B). Moreover, stressed *Tcf4*tg mice achieved a lower learning performance than stressed wildtype mice, indicating more severe deficits in learning flexibility. Similar deficits were observed when we continued altering corner assignments every 24 h for each animal over 6 days (serial reversal learning). Both genotype and environmental insult were found to decrease serial reversal learning performance [Figure 2C; G: *F*_(1,49)_ = 7.18; *p* = 0.034; E: *F*_(1,49)_ = 29.8; *p* = 4.47E-5]. Moreover, while we saw a more pronounced impairment in stressed *Tcf4*tg mice, suggesting an interaction of genetics and the environmental factor, this effect did not survive FDR adjustment [Figure 2C; G×E: *F*_(1,49)_ = 3.51; *p* = 0.0674; *p* adjusted = 0.185]. Notably, in a repeated measures two-way ANOVA, where we tested for pattern differences of the learning curve, the interaction of genotype and environment was also suggested but not significant, as well [Supplementary Figure 1; G×E: *F*_(1,57)_ = 3.53; *p* = 0.065]. Although this result should be treated with caution, because it was not adjusted for multiple comparisons and its equivalent in the main analysis pipeline did not survive the adjustment, together these results are suggestive for G×E interactions. The animal numbers in this pilot study were probably too low to provide the statistical power required for the large variable number of full behavioral phenotyping.

We further assessed fear memory function, i.e., the animal's ability to associate an aversive with a neutral stimulus when paired in a Pavlovian conditioning paradigm. We paired a visual context and an auditory cue with electric foot shocks and tested animals on two consecutive days for freezing behavior as a measure of fear. Stressed wildtype mice had a reduced freezing behavior in the contextual fear memory test 24 h after conditioning compared to non-stressed wildtype mice (Figure 2D). Comparing the two *Tcf4*tg groups, we further found that impairment of contextual fear memory was substantially enhanced in *Tcf4*tg mice exposed to psychosocial stress (Figure 2D). This is supported by a significant main effect of genotype (G) and environmental intervention (E) in a two-way ANOVA. Similar results were obtained for cued fear memory. The two-hit group of stressed *Tcf4*tg mice showed strongly reduced freezing behavior as compared to the healthy controls as well as both one-hit groups (Figure 2E). The absence of statistical interaction between the genetic and the environmental factor suggests a purely additive effect on contextual and cued fear memory.

In summary, these data suggest that psychosocial stress enhances cognitive deficits of *Tcf4*tg mice, especially in tasks assessing learning flexibility and strategy as well as fear memory.

#### Sensorimotor Systems

The sensorimotor gating network filters task-irrelevant information or stimuli and suppresses responses to them. To capture disturbances of sensorimotor gating, we performed the pre-pulse inhibition (PPI) test in our animals. Since patients suffering from schizophrenia often display deficits in PPI, this test offers high translational value and face validity (Greenwood et al., 2016).

We saw a decreased basic startle response in *Tcf4*tg mice, but no effect of psychosocial stress (Figure 2F). When assessing PPI with different pre-pulse sound levels, again we observed no differences between the two wildtype groups. The startle response in *Tcf4*tg mice, however, was increased compared to wildtype groups, independent of the environmental factor (Figures 2G–I). Interestingly, we found two potential subpopulations in the no-stress *Tcf4*tg group as well as three outliers with reduced PPI in the stressed *Tcf4*tg group at a pre-pulse level of 70 dBA. This might indicate that the mild overexpression of *Tcf4* causes miswiring of the PPI-relevant circuit to a variable degree.

To summarize, sensorimotor gating is solely affected by *Tcf4* gain of function. Psychosocial stress does not influence performance of wildtype nor *Tcf4*tg mice in a PPI test.

### Psychosocial Stress Impacts Parameters of Positive and Negative Valence Systems

#### Positive Valence Systems

**Figure 3:**
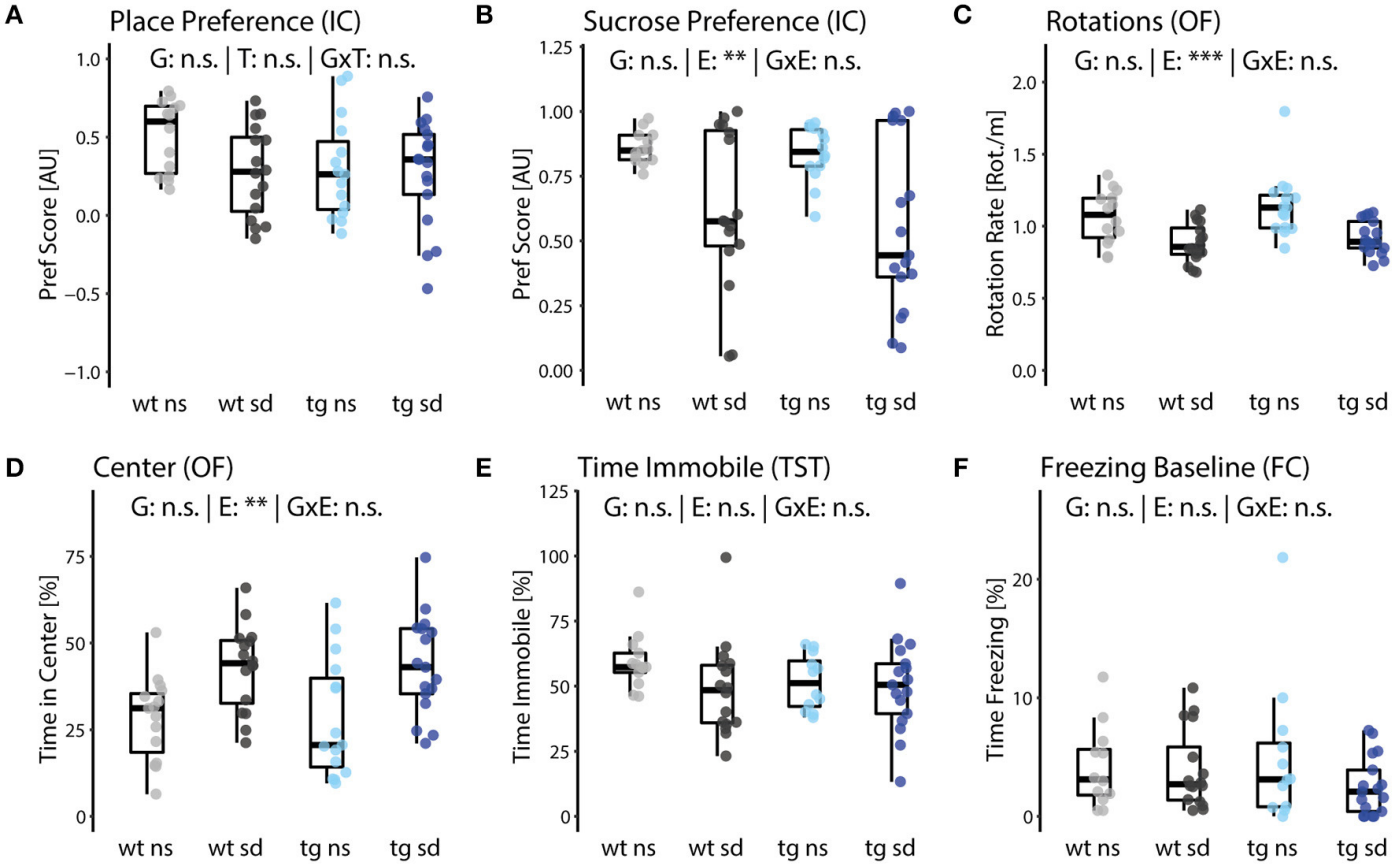
Impact of psychosocial stress on positive and negative valence systems. **(A)** Positively reinforced spatial learning (place learning) in the IntelliCage showed no significant difference between groups. **(B)** Preference for sucrose water and **(C)** frequency of directional change (rotations per distance traveled) in the open field test are reduced in the sd groups of both genotypes [E: **(B)** *F*_(1,49)_ = 12.6, *p* = 0.0043; **(C)** *F*_(1,49)_ = 19.32, *p* = 6.8E-4]. **(D)** Animals of the sd groups spent relatively more time in the brightly lit center of the open field arena [E: *F*_(1,49)_ = 12.5, *p* = 0.004]. **(E)** Struggling behavior measured in the tail suspension test and **(F)** general freezing behavior in a novel environment (fear conditioning box) did not differ between groups. Data are shown as box plots with whiskers extending to no more than 1.5-fold IQR; **p < 0.01, ***p < 0.001, n.s., not significant; *p*-values are FDR-corrected and refer to Wilk's lambda testing two-way ANOVA; *n* = 15/17/15/17. G, genotype term; E, environment term; G×E, interaction term.

As described for learning flexibility, animals were subjected to a place learning paradigm in the IntelliCage. Positively reinforced spatial learning was assessed by a preference score reflecting the success rate. Learning performance did not differ between groups (Figure 3A).

The learning paradigms were followed by a sucrose preference test to assess anhedonia in mice. For this paradigm, we replaced one of the two bottles in each corner with sucrose solution as a highly rewarding incentive and opened all doors for a 24-h period. Preference for the sucrose solution was quantified with a preference score, where positive values indicate preference while negative values represent avoidance, similar to place learning. While sucrose preference did not vary between genotypes, it did differ between non-stressed and stressed mice. We again observed a higher variance of responsiveness of stressed animals, which indicates a pronounced heterogeneity in the susceptibility to stress-induced behavioral alterations in groups of mice (Figure 3B).

#### Negative Valence Systems

In the open field test, animals were placed into white cubic boxes open at the top and with bright exposure as compared to the animal's home cages or the Y-maze. The frequency of directional changes (rotations) while ambulating inside the box, normalized to the total distance traveled, was used as an indicator of novelty or anxiety induced hyperactivity. Rotation rate was reduced for both groups exposed to psychosocial stress (Figure 3C). These groups also spent more time in the bright center of the test arena, suggesting less anxiety, while *Tcf4* overexpression did not affect either parameter (Figure 3D).

The time mice spend immobile in the tail suspension test is an indicator of acceptance of aversive situations. We did not observe statistically significant differences between any of the groups although variance was higher for the stressed groups, which again points to different levels of susceptibility to stress in groups of mice (Figure 3E).

We also measured freezing behavior in the enclosures of fear conditioning boxes before actual conditioning as a proxy of anxiety in a novel environment. No group differences were detected (Figure 3F).

In summary, we show that positive and negative valence systems are affected by psychosocial stress, independent of *Tcf4* overexpression.

### Psychosocial Stress Increases Novelty-Induced but Not General Locomotor Activity

#### Arousal and Regulatory Systems

**Figure 4:**
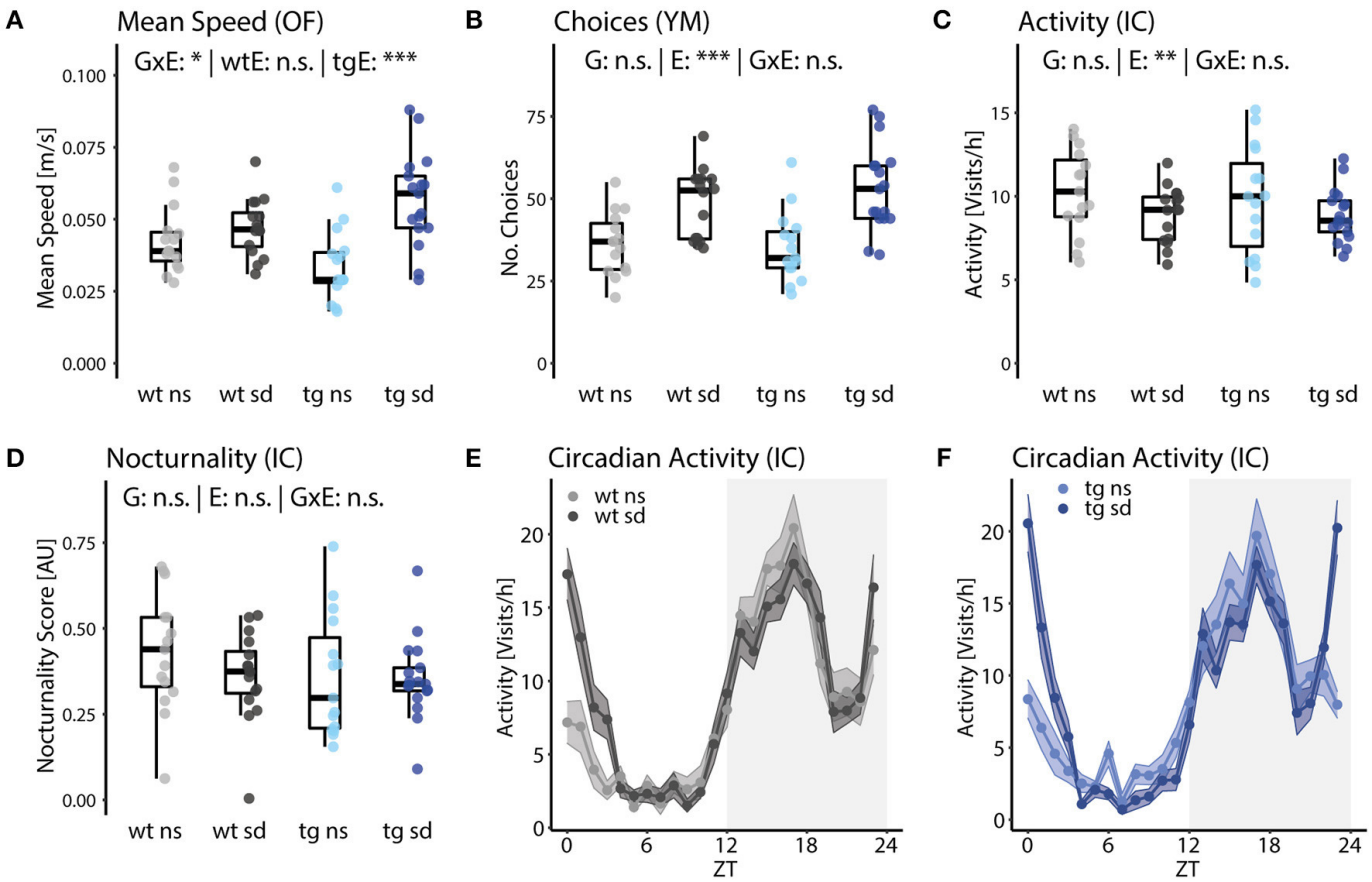
Psychosocial stress increases novelty-induced but not general locomotor activity. **(A)** Mean speed in the open field test revealed a statistically significant interaction of genotype and environment [G×E: *F*_(1,49)_ = 8.4, *p* = 0.020]. A subsequent one-way ANOVA for each genotype shows that social defeat induces increased mean speed only for tg animals [tgE: *F*_(1,30)_ = 20.4, *p* = 9.0E-5]. **(B)** The number of choices in the Y-maze test were significantly increased by social defeat, as well, without a significant interaction with genotype [E: *F*_(1,49)_ = 23.0, *p* = 2.8E-4]. **(C)** Overall activity in the IntelliCage over 5 days was found to be reduced in stressed mice [E: *F*_(1,49)_ = 12.5, *p* = 0.0043]. **(D–F)** Nocturnality of activity in the IntelliCage did not differ significantly between groups. However, a repeated-measures ANOVA of hourly bins revealed that circadian allocation of activity was influenced by social defeat [ExZT: *F*_(23,1,380)_ = 9.05, e = 0.52, p[HF] = 2.52E-16]. Data are shown as box plots with whiskers extending to no more than 1.5-fold IQR; *p < 0.05, **p < 0.01, ***p < 0.001, n.s., not significant; *p*-values are FDR-corrected and refer to Wilk's lambda testing two-way ANOVA; *n* = 15/17/15/17. G, genotype term; E, environment term; G×E, interaction term; ExZT, Interaction of environment and zeitgeber time; wtE, effect of environment in wt mice; tgE, effect of environment in tg mice.

Moreover, PsyCoP includes several measures of arousal and circadian regulation:

Ambulation in the open field measures the response to a novel environment as the mean speed, i.e., the total distance traveled over the time of the experiment. While we did not observe differences between the two non-stressed groups, social defeat induced hyperactivity in *Tcf4*tg mice, but not wildtypes in the open field test (Figure 4A). Similar results were obtained from the Y-maze test, where we assessed the number of arm choices as a measure for novelty-induced activity without the aversion of brightness in the open field test (Figure 4B). Such overlapping measures between tests increase the robustness of our test battery and can compensate for potential confounding factors. Notably, the interaction of genetic (G) and environmental (E) factor for mean speed is statistically significant [Figure 4A; G×E: *F*_(1,49)_ = 8.41; *p* = 0.048] and the corresponding one-way ANOVA for each genotype revealed a significant effect of social defeat in *Tcf4*tg mice but not wildtypes [Figure 4A; E: tgE: *F*_(1,30)_ = 20.4; *p* = 8.99E-5; wtE: *F*_(1,29)_ = 1; *p* = 0.256]. Therefore, we conclude that novelty-induced hyperactivity is driven by *Tcf4* gene dosage and psychosocial stress in adolescence in a synergistic manner.

To separate novelty-induced from general activity, we continuously monitored overall activity in the IntelliCage over several days. The frequency of corner visits was reduced in stressed mice compared to no stress controls, suggesting that psychosocial stress in adolescence induces novelty-induced hyperactivity while it might reduce general activity in adult mice [Figure 4C; E: *F*_(1,49)_ = 12.5, *p* = 0.0043]. Additionally, circadian activity amplitude was assessed by calculating a nocturnality score, i.e., the preference for nighttime vs. daytime activity. A circadian phenotype might reduce the nocturnality score, however, we did not observe differences between groups (Figure 4D). However, possible differences might have been masked by the 12-h light-dark cycle during the whole experiment, for the circadian activity profile did reveal that the circadian allocation of activity, i.e., the change in activity throughout 1 day/night, was indeed altered by psychosocial stress [Figures 4E,F; ExZT: *F*_(23,1,380)_ = 9.05, e = 0.52, p[HF] = 2.52E-16]. The main contributor to this effect is probably the sudden spike in activity of the stressed mice at dawn.

In summary, general activity was reduced in groups subjected to social defeat and circadian activity preference did not differ between groups, whereas novelty-induced activity is increased in mice previously exposed to psychosocial stress. *Tcf4* gain of function alone does not affect the arousal and regulatory traits assessed in our testing pipeline. Notably, we found that hyperactivity in the open field test is explained by an interaction of *Tcf4* gain of function and psychosocial stress.

### The Two-Hit Disease Model Clusters Separately From Healthy Controls

#### Visualization of Neurocognitive Profiles in a Heatmap

**Figure 5:**
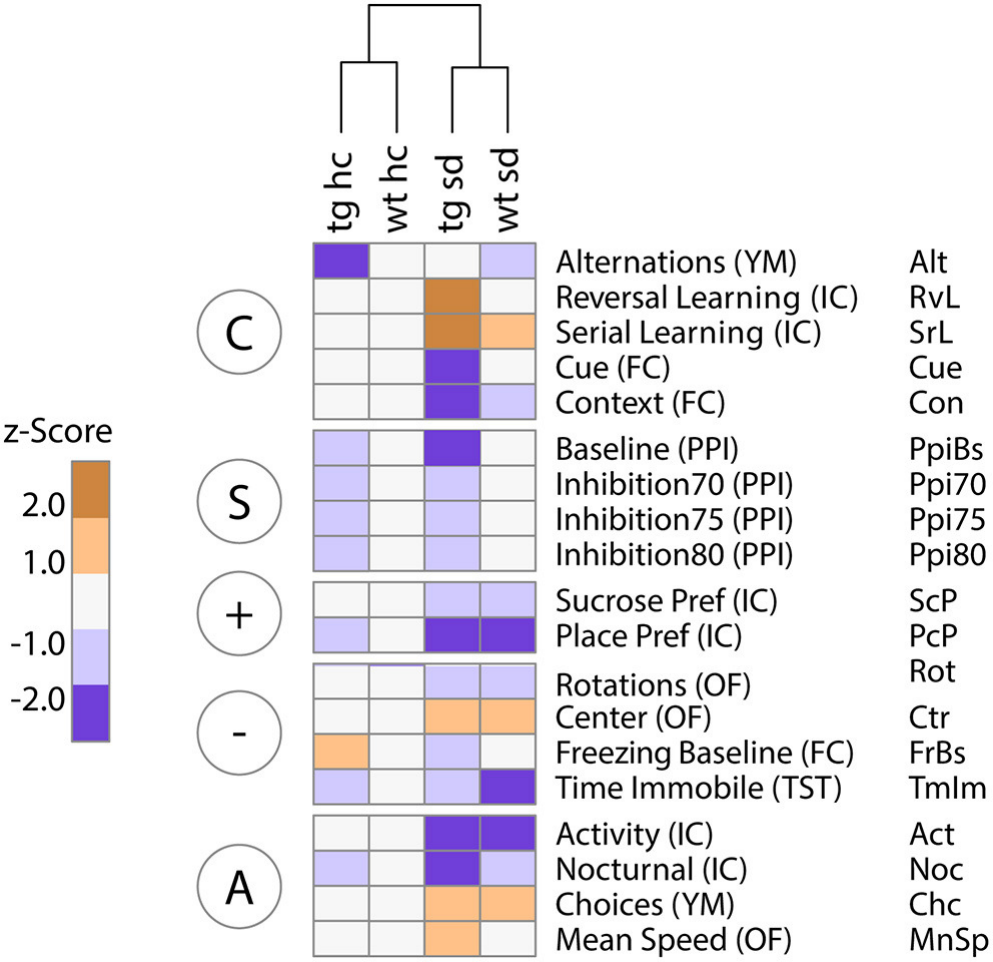
RDoC grouping of *z*-scored data reveals domain-specific differences in the contribution of *Tcf4* gene dosage and psychosocial stress to behavioral and cognitive dysfunction. Unsupervised hierarchical clustering of group means of standardized (*z*-scored) behavioral data clusters primarily by environmental stress. The heatmap highlights changes relative to the healthy control group [wildtype no stress control (wt ns)]. The most prominent effects are seen for the two-hit disease group *Tcf4*tg mice subjected to social defeat during adolescence (tg sd). Particularly, deficits in the cognitive domain (“C”) are dependent on both genetic and environmental impact, in contrast to more generally stress-dependent abnormalities in the positive (“+”) and negative (“–”) valence as well as the arousal/regulation domain (“A”). The sensorimotor gating system (“S”) clearly separates animals based on *Tcf4* gene dosage only. Column blocks are defined by RDoC domains; *n* = 15/17/15/17.

In order to visualize the neurocognitive profile of different groups of animals, we created a heatmap. All data acquired in the behavioral tests were *z*-transformed and centered on the group means of the healthy control group, wt ns. Variables were grouped according to their corresponding RDoC behavioral domains (Figure 5). This heatmap offers a fast, intuitive, easy to understand first overview of the results.

Hierarchical clustering separated the four groups primarily by environmental condition (ns vs. sd), underlining the stronger impact of psychosocial stress on cognition and behavior compared to mild *Tcf4* overexpression (Figure 5). For the cognitive system (C), stressed *Tcf4*tg mice showed a clear separation from healthy controls, while for the sensorimotor system (S), *Tcf4*tg differ from wildtype mice independent of the environmental (Figure 5). For the positive valence system (+), place preference clearly separated all other groups from the wildtype no stress control (wt ns) (Figure 5). However, because of relatively high data variation no statistical significance for place preference was reached in a univariate ANOVA (Figure 3A). For the negative valence system (–), the time spent immobile in the tail suspension test was lower for all groups compared to wt ns. However, for the same reasons, this effect did not reach significance in a univariate ANOVA (Figure 3E).

In summary, we visualized distinct behavioral profiles of our two-hit disease model with all groups in a single, easily interpretable heatmap. It should be noted that not all visible differences are significant in standard statistical tests. Therefore, the presented heatmap should be regarded as a “soft” visualization tool suitable for easy and intuitive comprehension of complex data.

#### Dimension Reduction by Canonical Discriminant Analysis

**Figure 6:**
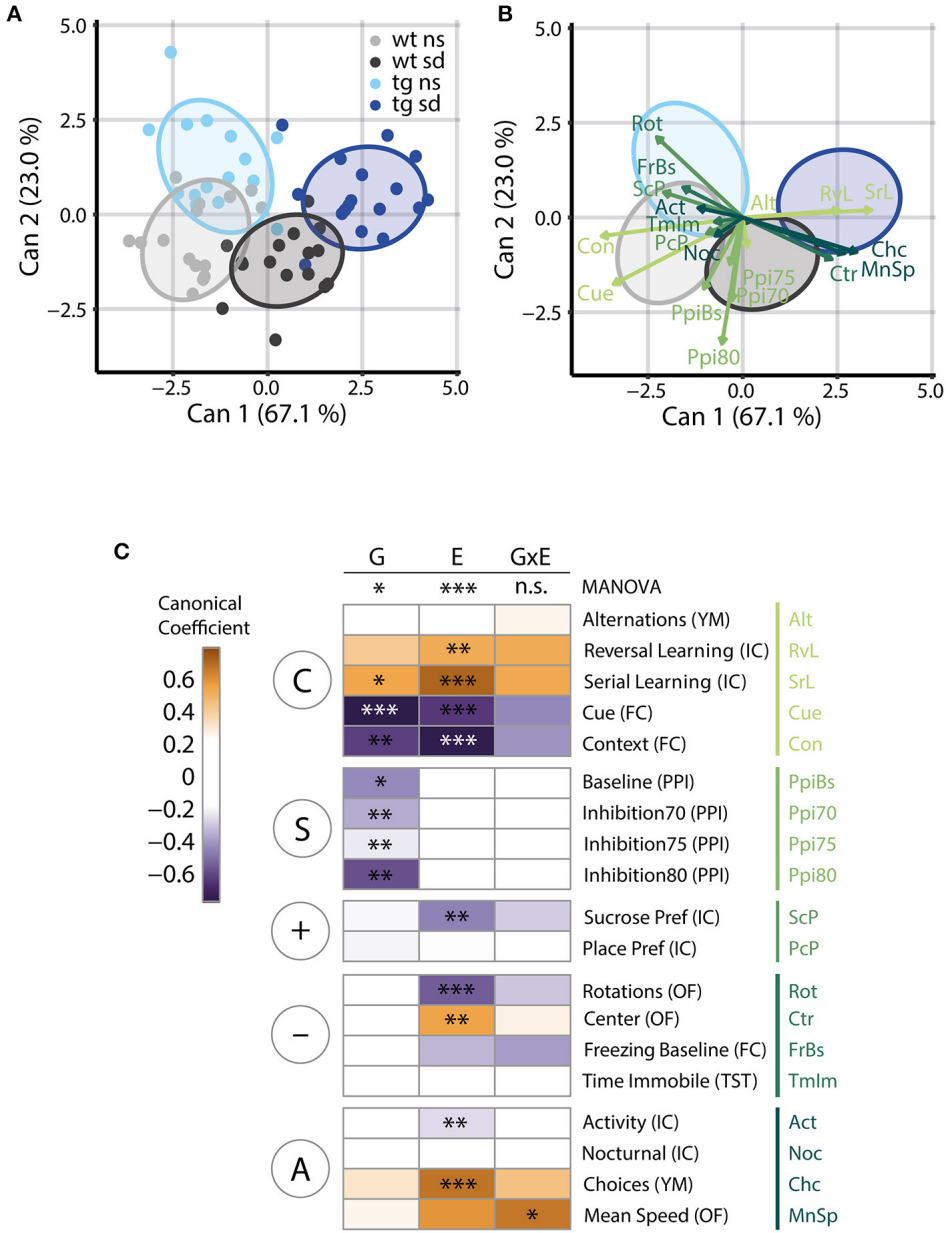
Canonical discriminant analysis reveals striking group differences modulated by genetic and environmental factors. **(A)** Canonical Discriminant Analysis (CDA) of the collective data reveals that the stronger canonical component 1 (Can1), which explains 67.1% of the total canonical correlation, separates the datapoints along the environmental factor, while the second component, amounting to 23.0% of canonical correlation, separates the datapoints according to *Tcf4* gene dosage. Ellipsoids visualize 75% coverage of each group and each animal is depicted as correspondingly colored dot; *n* = 15/17/15/17. **(B)** The CDA dimension plot depicted without individual data points but with vector representations of canonical coefficients instead, indicates the contribution of single variables to the structure of each canonical component. Several measures of cognitive ability influence both components, among them contextual and cued fear memory, while novelty-induced activity, as measured with mean speed in the open field and choices in the Y-maze, mainly separate psychosocially stressed animals from controls. Sensorimotor gating disturbance appears to be a trait of *Tcf4* gain of function, again. **(C)** A heatmap displaying the contribution of each variable to the separation along each term of the linear model of the CDA in a color code shows RDoC domain-specific differences in the contribution of gene and environment to the observed group separation. The asterisks indicate the adjusted *p*-value of significant terms of the univariate ANOVAs displayed in Figures 2–4. In summary, the MANOVA reveals a significant effect of gene dosage (“G”), in the form of *Tcf4* overexpression, on the neurocognitive profile of our model [*F*_(19,31)_ = 2.52, *p* = 0.011] as well as a clear effect of the environment (“E”), i.e., social defeat [E: *F*_(19,31)_ = 7.83, *p* = 3.32E-7]. These effects appear to be additive, but overall, no statistical interaction of gene and environment (“G×E”) was found [*F*_(19,31)_ = 1.41, *p* = 0.194]. Again, grouping of the variables in RDoC domains shows a strong influence of social defeat on positive (“+”) and negative (“–”) valence systems as well as regulatory and arousal systems (“A”). *Tcf4* gene dosage has a major impact on sensorimotor systems (“S”). While G has no significant influence on arousal by itself, the G×E interaction is significant in Mean Speed. Both factors contribute to the separation of groups by their cognitive performance (“C”). Column blocks are defined by RDoC domains. *n* = 15/17/15/17. The F statistics given refer to a Wilk's lambda approximation based two-way MANOVA.

Dimension reduction is commonly used to condense high-dimensional data without losing information. The most common procedure for dimension reduction is principal component analysis (PCA). However, in the context of the PsyCoP approach, we used Canonical Discriminant Analysis (CDA) to find linear combinations of variables (canonical components) that, in contrast to PCA, are optimized for separation of the experimental groups. From these, we calculated canonical scores for each animal and visualized them in a dimension plot (Figure 6A) and the structure of these components either as vectors (Figure 6B) or in a heatmap (Figure 6C). The dimension plots offer a condensed representation of the phenotypic space, in which all four groups are distinguishable, while visualization of the canonical coefficients additionally allows to judge the importance of single variables to group segregation. With the help of the dimension plots, a researcher can see how the groups are distributed in phenotypic space, while either method of visualization of the canonical coefficients tells at one glance, which traits are impacted in the disease model under investigation and to which research domain they belong. This gives the researcher a clear overview over the neurocognitive and behavioral profile at one glance.

In our two-hit model, the first and strongest canonical component accounted for 67.1% of canonical correlation and distinguished groups by the environmental factor, while the second component, accounting for 23.0%, separated groups by genotype. Notably, the healthy control (wt ns) and the disease model (tg sd) showed no overlap. We also tested principal component analysis (PCA) as method of dimension reduction, but CDA gave better group separation as expected (Supplementary Figure 2).

Furthermore, the structure of each component can be used to study the contribution of single variables to the separation of groups along the respective dimension. Vector representations of the canonical coefficients visualize the contribution of each behavioral test variable to each canonical component (Figure 6B). The absolute values of these coefficients are weights for the contribution of the respective variable to the canonical score. As the canonical score represents the optimal linear combination of variables for group separation, the coefficients indicate the contribution of the respective parameter to group separation. In short, the length of the vectors shown in Figure 6B represent the importance of the corresponding variable for the segregation of groups in phenotypic space.

While cognitive traits seemed to influence both components and clearly separated stressed *Tcf4*tg mice from the healthy control, sensorimotor gating separated groups almost exclusively by *Tcf4* overexpression. Novelty-induced activity separated groups by the environmental factor.

In order to further analyze the influence of *Tcf4* overexpression and psychosocial stress, we analyzed the CDA of each term of the multivariate linear model individually. We visualized the canonical coefficients of the resulting canonical scores in a heatmap with the respective univariate ANOVA results indicated on top (Figure 6C).

A multivariate ANOVA of all variables confirmed that both the genotype and the environmental factor affect the behavioral and cognitive phenotype [G: *F*_(19,31)_ = 2.52; *p* = 0.011; E: *F*_(19,31)_ = 7.83; *p* = 3.32E-7]. Of note, the interaction term was not statistically significant [G×E: *F*_(19,31)_ = 1.41; *p* = 0.1935].

As seen in the *z*-score based visualization, cognitive system measures separated groups both by genotype and by environmental factor. In contrast, pre-pulse inhibition as an RDoC element of sensorimotor systems distinguished groups based on genotype only (Figure 6C).

Positive and negative valence systems set apart stressed from control mice. While novelty-induced agitation as an RDoC element of the arousal/regulatory domain was mostly influenced by psychosocial stress, it also contributed to the separation of genotypes. Notably, mean speed was the only statistically significant interaction of the genetic and the environmental factor surviving FDR adjustment (Figure 6C; Supplementary Table 3).

In summary, CDA allows detecting relevant behavioral alterations between the experimental groups. For the *Tcf4*tg mouse model, it identifies the cognitive, sensorimotor, and arousal system measures as the strongest contributors to group differences. In addition, it validates findings from the simpler group comparisons and heatmap analyses, while revealing the deeper structure of our multi-dimensional data set.

## Discussion

In this study, we developed the PsyCoP workflow and applied it to the two-hit psychiatric disease mouse model of *Tcf4* overexpression in combination with psychosocial stress in adolescence.

PsyCoP comprises an arrayed battery of 10 different behavioral tests generating 19 partially redundant behavioral parameters which were grouped into five research domains providing a rich behavioral repertoire of relevance for psychiatric diseases. The complete run time of all tests of the pipeline adds up to 5 weeks and when combined with a chronic social defeat paradigm that lasts 3 weeks, PsyCoP takes in total only 2 months. Of these 10 behavioral tests, 4 are conducted in the IntelliCage and are fully automatized because of the telemetric recordings that are independent of the daytime. The readout of all other tests is automatized by applying either video tracking or electronic recordings in case of the startle response measurements. Nonetheless, animals are individually handled and leave their home cage for the duration of the test, which is a limitation of the current setup. Thus, we refer to the PsyCoP platform as partially or semi-automatized solution. To overcome this limitation, handling could be further reduced in the future by connecting different types of cages into a so-called PhenoWorld setup, where transponder-responsive gates allow voluntary or controlled access of individual animals to different parts of the arena. Thereby, different automated home cage systems such as conditioning boxes and the digital ventilated cages from Tecniplast could be combined with IntelliCages as suggested in a recent review (Voikar and Gaburro, 2020). Such systems to completely automatize the analysis of behavior in a more naturalistic environment have already been developed for mice and rats and allow for simultaneous monitoring home cage behavior, locomotor activity, hedonic and social behavior as well as cognition (Castelhano-Carlos et al., 2014; Torquet et al., 2018). Further, the PsyCoP platform should be amended by testing of social behavior to completely cover the main research domain categories for psychiatric disorders as suggested by the NIMH (NIMH RDoC Matrix; Insel et al., 2010). Since alterations of the sleep-wake architecture are a critical hallmark of psychiatric disorders, telemetric EEG-recordings or other physiological parameter could be implemented in the future to further expand the behavioral repertoire of PsyCoP (Gaburro et al., 2011; Camp et al., 2012).

Severe cognitive deficits of stressed *Tcf4*tg mice are apparent from our RDoC categorized heatmap. The genotype and environment interaction term of most individual univariate ANOVAs did, however, not reach significance when corrected for comparisons across all variables. Although being a standard statistical procedure, stringent false discovery rate adjustments are generally not applied in the field of behavioral neuroscience but would certainly increase the robustness of conclusions. From our analysis, we conclude that group sizes should be more than 20 animals per group to detect changes of smaller effect size more robustly.

We showed that sensorimotor gating abilities are strongly influenced by *Tcf4* gene dosage, reproducing and validating our previous findings (Brzózka et al., 2010). Additionally, the factor psychosocial stress affects arousal and regulation behavior. Cognitive traits, particularly deficits in flexibility learning as assessed in an automated reversal task in the IntelliCage, clearly separate the two-hit disease model from healthy controls. This is in agreement with independent Morris water maze results from our lab, demonstrating the robustness of the detected phenotype (Badowska et al., 2020).

The dimension plots derived from *canonical discriminant analysis* (CDA) show a clear separation of the experimental groups, particularly along the environmental risk factor, but also along the genetic factor axis. Moreover, the disease model group (*Tcf4* × sd) clustered separately from the healthy control group, again emphasizing its distinct phenotype.

Overlapping measures from different tests contribute similarly to the latent variables as illustrated by vector representations in our dimension reduced visualizations (Figure 6), confirming the robustness of our pipeline.

With PsyCoP, we do not intend to model the entire symptom complex of schizophrenia and other psychiatric disorders simultaneously. We rather aim at identifying distinct endophenotypes, which can then be addressed individually in subsequent streamlined compound screens and treatment trials. These individual endophenotypes might be prevalent in different subgroups of patients and targeting them individually could lead to more effective treatments in the context of precision medicine (DeLisi and Fleischhacker, 2016). One endophenotype of high translational value is PPI, as disturbed sensorimotor gating is even found in unaffected relatives of schizophrenia and bipolar patients (Giakoumaki et al., 2007; Greenwood et al., 2016). Our 2 × 2 factorial analysis revealed that the PPI endophenotype observed in *Tcf4*tg mice is independent of environmental stress. It likely reflects wiring deficits established early in neuronal circuit formation. It will be interesting to see whether compounds can be identified that are capable of modulating such a “wiring-deficit” without undesirable side effects. Moreover, our two-hit *Tcf4*tg/social defeat mouse model addresses deficits in cognitive flexibility, a common feature of schizophrenia that still lacks effective pharmacological treatment options (Goff et al., 2011; Falkai et al., 2015).

We are currently using sensorimotor systems and cognition as selected domains for testing compounds in treatment trials. Additionally, we are currently incorporating additional RDoC dimensions such as the social domain. The current test battery is restricted to male mice because of the social defeat stress paradigm. It is known that the effects of stress in adolescence are sex dependent, so it would be important to be able to test both sexes. For that reason, we are evaluating other paradigms such as unpredictable chronic mild stress (Buhusi et al., 2017; Page and Coutellier, 2018).

Our new pipeline and analysis platform PsyCoP can be used to standardize data acquisition and analysis across different laboratories. We intentionally selected behavioral tests that are easy to perform, that are robust and that cover a broad spectrum of traits relevant in psychiatric disorders, while allowing a reasonably high throughput of disease models, as discussed in (Stephan et al., 2019). The analyses offer both, depth of insight as well as overview. It can be used to correlate psycho-affective endophenotypes with genotype and developmental or environmental factors. By placing all protocols and analysis scripts in the public domain, we hope that other research sites adapt this approach to their work.

In this study, we present PsyCoP, a standardized phenotypic behavioral profiling battery and data analysis pipeline. Overlapping measures from different tests as well as the analysis in the framework of the NIMH RDoC psychiatric classification scheme contribute to the robustness and predictive validity of our approach. We applied this workflow to the two-hit disease model of overexpression of the psychiatric risk gene *Tcf4* in combination with psychosocial stress during adolescences. Our data show that *Tcf4* overexpression and psychosocial stress synergistically affect cognitive traits and novelty-induced hyperactivity, while other behavioral domains are affected by single factors alone.

The PsyCoP workflow is well suited for two-hit models of psychiatric diseases and can guide phenotypic compound screens and preclinical drug development in areas of currently unmet therapeutic needs in the treatment of psychiatric diseases.

## Data Availability Statement

The datasets presented in this study can be found in online repositories. The names of the repository/repositories and accession number(s) can be found at: https://github.com/volkmannp/PsyCoP.

## Ethics Statement

The animal study was reviewed and approved by Regierung Oberbayern, Munich, Germany.

## Author Contributions

PV and MS performed tests and analyzed the data. SK contributed to the data analysis workflow by implementing the statistical tools in a FlowR bundle. NJ contributed conceptually, helped with the setup. MR devised the study and contributed to data analysis. All authors contributed to writing of the manuscript.

## Conflict of Interest

The authors declare that the research was conducted in the absence of any commercial or financial relationships that could be construed as a potential conflict of interest.
