# Supplementary material for: PsyCoP – A Platform for Systematic Semi-Automated Behavioral and Cognitive Profiling Reveals Gene and Environment Dependent Impairments of Tcf4 Transgenic Mice Subjected to Social Defeat
Source: Front Behav Neurosci. 2021 Jan 14;14:618180. doi: 10.3389/fnbeh.2020.618180 (PMC7841301; doi:10.3389/fnbeh.2020.618180)
Supplement: Supplementary file 1 [file Image_1.pdf]

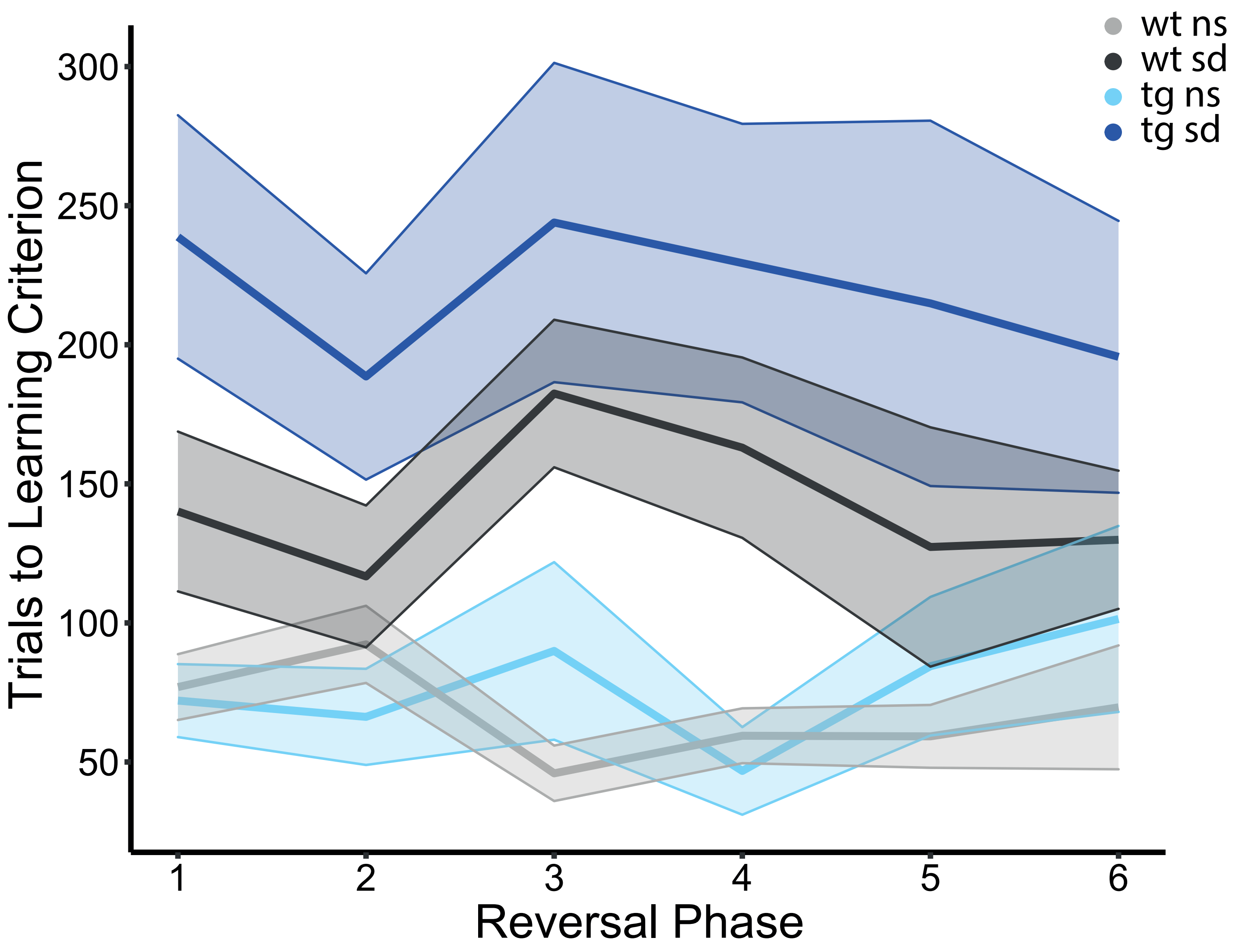

Suppl. Figure 1. Learning Performance of Two-Hit Mice Is Worse Than Healthy Control Throughout Serial Reversal Learning.

The graph displays the mean number of trials needed to reach the learning criterion determined by Sequential Probability Ratio Testing (SPRT) as line with the standard error of the mean indicated as ribbon plotted against the six reversal phases. In case a mouse did not reach the learning criterion within the 24 hours of a phase, it was included in the analysis with the total number of trials, underestimating the number of trials needed. There is no significant effect within learning curves, probably due to a high variability between phases in individual animals (Phase: F(5, 290) = 0.341; e = 0.595; p[HF] = 0.794). We found a significant main effect of genetic and environmental factor (G: F(1, 58) = 6.86, p = 0.0112; E: F(1, 58) = 38.7, p = 5.87E-8; GxE: F(1, 58) = 3.53, p =0.065), confirming the effect suggested by the main analysis shown in Figure 2C. The dataset was analysed in a repeated measures two-way ANOVA with Type 2 Sum of Squares with subsequent Huynh-Feldt correction after the assumption of variance homogeneity was found to be violated in a Maulchy's test for sphericity (W = 0.212, p = 1.50E-12). Normality of the model residuals was tested with an E-test for normality (N = 61; R = 1000; E = 0.975, p = 0.052). Subsequent one-way ANOVAs found no significant within subject effect (Phase: wt: F(6, 174) = 0.284; p[HF] = 0.231; tg: F(6, 168) = 1.49 , p[HF] = 0.307), but a significant main effect of the environmental factor only in Tcf4tg mice (E: wt: F(1, 29) = 0.284, p = 0.598; tg: F(1, 28) = 14.8, p = 6.32E-4). More details on the statistics can be found in Supplementary Table 3.
