# Supplementary material for: PsyCoP – A Platform for Systematic Semi-Automated Behavioral and Cognitive Profiling Reveals Gene and Environment Dependent Impairments of Tcf4 Transgenic Mice Subjected to Social Defeat
Source: Front Behav Neurosci. 2021 Jan 14;14:618180. doi: 10.3389/fnbeh.2020.618180 (PMC7841301; doi:10.3389/fnbeh.2020.618180)
Supplement: Supplementary file 2 [file Image_2.pdf]

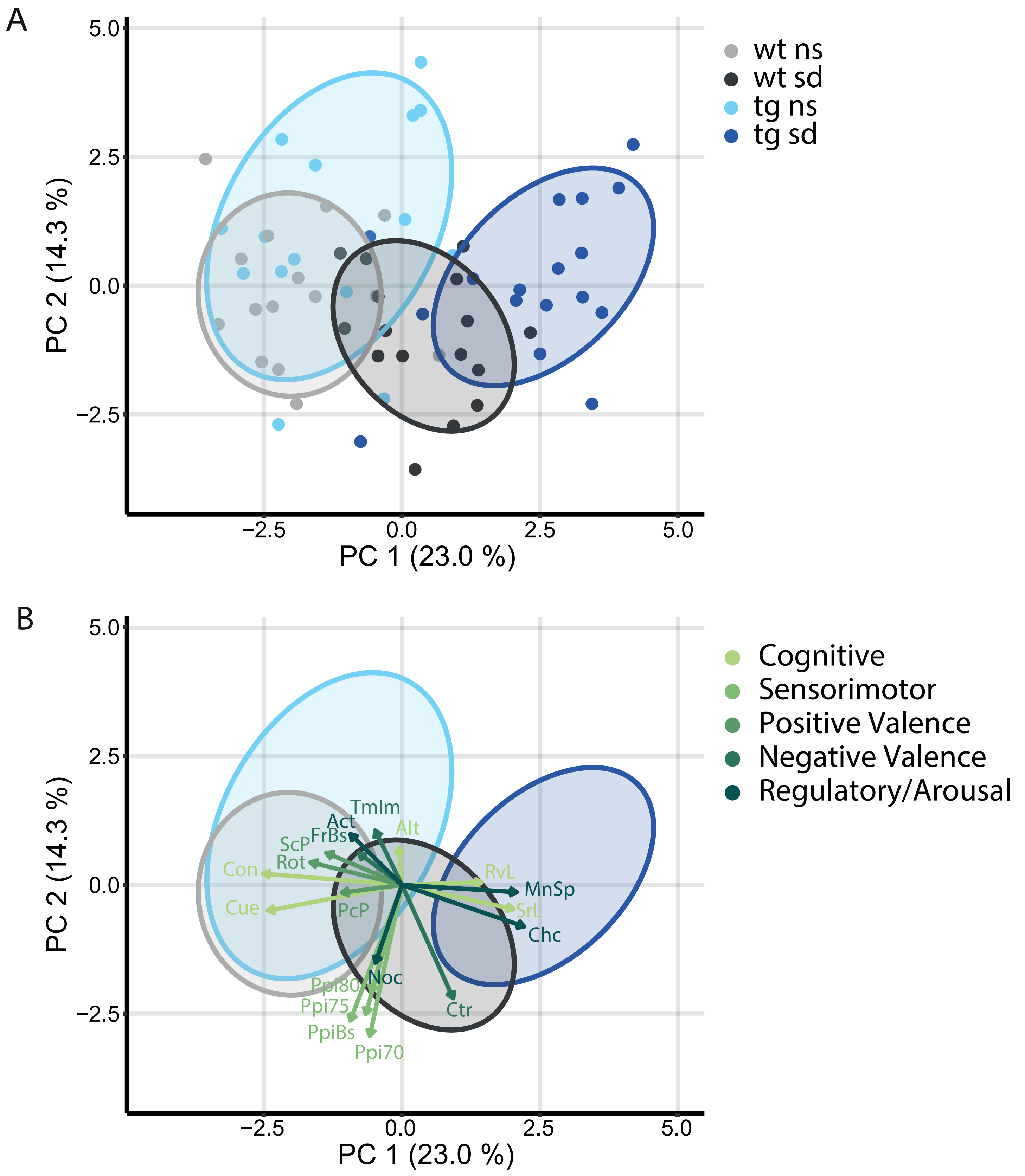

Suppl. Figure 2. PCA separates test groups by genetic and environmental conditions.

(A) Principal Component Analysis (PCA) reveals that Principal Components (PC) 1 and 2 separate all groups, especially environmental conditions, with a large overlap of wt hc and tg hc, indicating only small differences in their phenotypic space. Both principal components appear to be equally informative, PC1 explaining 23.0 % of variance, PC2 14.3 %. Ellipsoids visualize 75 % coverage of each group; each animal is depicted as correspondingly colored dot; n = 15/17/15/17. (B) Within PCA, learning abilities, conditioned fear memory and (novelty-induced) activity separate groups by both factors, but with stronger impact of environmental disposition, whereas the sensorimotor domain clearly distinguishes the genotype. PCA is plotted with centered vectors indicating the contribution of single variables to each dimension/principal component. These vectors are colorcoded to indicate the research domain the respective variables belong to.
