# Supplementary material for: PsyCoP – A Platform for Systematic Semi-Automated Behavioral and Cognitive Profiling Reveals Gene and Environment Dependent Impairments of Tcf4 Transgenic Mice Subjected to Social Defeat
Source: Front Behav Neurosci. 2021 Jan 14;14:618180. doi: 10.3389/fnbeh.2020.618180 (PMC7841301; doi:10.3389/fnbeh.2020.618180)
Supplement: Supplementary file 3 [file Image_3.pdf]

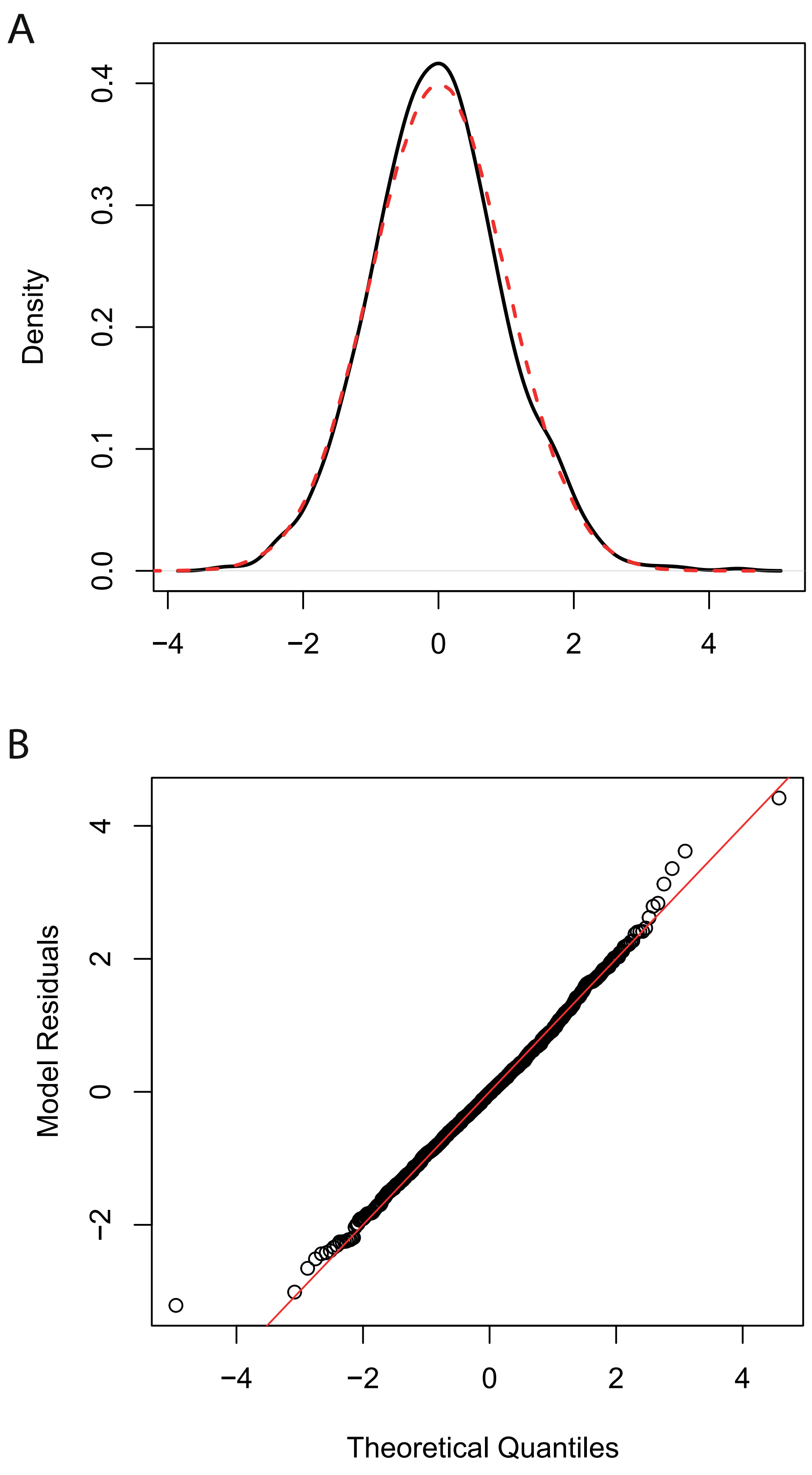


Suppl. Figure 3. Visual controls confirm normality of the MANOVA model's residuals for the behavioral dataset

Before applying ANOVA statistics to the behavioral dataset the assumption of normality was checked on the residuals of the multivariate linear model used for downstream analysis. (A) Shows a density plot of the scaled residuals of the fitted linear model overlayed with a normal distribution as a dashed red line (mean = 0; sd = 1). (B) displays a quantile-quantile plot comparing the scaled model residuals with a normal distribution. The red line indicates identity of the distributions. Both plots indicate minor deviations from normality, which were not statistically significant in an E-test for multivariate normality (N = 53; dims = 19; R = 1000; E = 2.2832; p = 0.100).
