# Supplementary material for: PsyCoP – A Platform for Systematic Semi-Automated Behavioral and Cognitive Profiling Reveals Gene and Environment Dependent Impairments of Tcf4 Transgenic Mice Subjected to Social Defeat
Source: Front Behav Neurosci. 2021 Jan 14;14:618180. doi: 10.3389/fnbeh.2020.618180 (PMC7841301; doi:10.3389/fnbeh.2020.618180)
Supplement: Supplementary file 4 [file Presentation_1.pdf]

Supplementary Descriptions of Experimental Procedures

At least 10 minutes before each testing session, animals were transported to the experiment room in their home cages for habituation. Experimental mice were taken out of their cages with clear polycarbonate tunnels to avoid the stress induced by classical tail handling (Hurst and West, 2010). Experiments were carried out by the same experimenter throughout all tests for every mouse.

Social groups of animals were put together in their home cage as early as possible, typically between postnatal day 21 and 28.

All test equipment was cleaned using SDS solution and then ethanol before and after usage to remove olfactory cues if not stated otherwise. Animals were exposed to the following tests consecutively.

1 Social Defeat

Social defeat in the context of a resident intruder paradigm was used to expose animals to psychosocial stress in their adolescence.

For the training of resident mice, we put together FVB/N male mice, 45- to 70-week-old, with adult female mice. Residents stayed with their dams for 3 days. After separation from their dam, attack latency of the potential residents was assessed with 8 weeks old male C57Bl/6N wildtype mice (not included in behavioral experiments). FVB/N mice with attack latencies of less than 20 seconds were included as residents in later social defeat tests. Residents were single housed for them to establish their own undisputed territory within their home cage.

On 21 consecutive days, starting at an age of 34 to 42 postnatal days, experimental mice of the social defeat group (intruders) were taken out of their home cage individually and introduced to a resident's home cage for a total of 21 sessions for each intruder test mouse. After the first physical attack run of a resident had occurred, animals were separated by a wire mesh cage (75 mm × 115 mm × 60 mm). At no time, animals suffered bite marks or any other signs of injury. The intruder remained underneath the meshed cage in the resident's cage with the resident present for another 60 minutes. Afterwards, animals were identified by their ear tags, their tails were marked with a color code and animals were put back into their home cages. They were kept in group housing conditions at all times. Metal cages were cleaned with water and ethanol before they were used again.

The session time was randomized daily between 7 a.m. and 7 p.m. Pairing of residents and intruders was pseudo-randomized in a rotation scheme to avoid repeated contacts of a given intruder mouse with the same resident. In this scheme, a given intruder mouse encountered the same resident only once in 12 days and no more than twice in total. Moreover, the temporal order of intruder cages was changed on each day.

Animals of the no stress control group were kept in their home cages during adolescence. Mice were taken out of the cages 3 times daily for the last 3 days of the social defeat period to accustom them to handling procedures. Animals exposed to social defeat were housed in different cages than control mice.

As social defeat served the purpose of exposing animals to psychosocial stress, no data was collected, and animals were only observed by the experimenter.

2 Open Field Test

Novelty-induced and spontaneous exploratory behavior was monitored at ZT2 (zeitgeber time; two hours after light onset/dawn) in an open field arena (50 cm x 50 cm x 50 cm white boxes open at the top) for 10 minutes. Mice were put into their respective box facing one of the four walls in the same orientation for every mouse. The videorecording started automatically after the experimenter had left the recording area. Distance travelled, maximum speed, time spent in predefined areas (e.g., center), number of (im)mobile episodes, time (im)mobile and the number of rotations were assessed with the behavioral tracking software ANY-maze (Stoelting, Wood Dale, IL, USA). From these parameters, we derived the variables mean speed (distance travelled / recording time), rotation rate (rotations / distance travelled) and center time. The center area was defined as the total area excluding the 5 cm strip next to the walls and the 10 cm square of each corner. Illumination during the test was set to approximately 1600 lux.

3 Y-Maze Test

Working memory capacity was assessed by quantification of spontaneous alternations in the Y-maze at ZT6. The Y-maze consists of three identical arms (A, B, C) in the shape of a “Y”. The test was conducted at 50-70 lux for 10 minutes. Spontaneous alternations describe the rate of full sequences of visits (choices) to each arm of the arena without repetition (e.g., A-B-C, B-A-C or B-C-A, but not A-B-A, C-B-C, or B-A-B). They were calculated as percentage with the following formula:

Alternations = sum(full sequences) / (choices - 2) * 100 %

Mice were put into arm A facing the center. Video tracking of the mouse with the ANY-maze software started automatically, after the experimenter had left the recording area.

4 IntelliCage System

The IntelliCage system (www.tse-systems.com/product-details/intellicage) is a commercial home cage-based monitoring and conditioning device. It consists of a frame put into type 4 Tecniplast cages. The frame is composed of four corners, each with a door on each of two sides. Mice could access a water bottle behind these doors, if opened. In case doors were closed, mice could open the doors (depending on the current paradigm) by disrupting a light barrier at a given door with a nosepoke under paradigm dependent conditions. Usually, only one mouse was present in a corner at a time. The registration of a known RFID in combination with a heat signal was defined as a visit. Visits to a corner, nosepokes and licks at water bottle nipples were monitored continuously throughout all IntelliCage experiments. Food was provided ad libitum in the middle of each cage.

Mice were introduced to the IntelliCage on the day of Y-maze-testing at ZT9. The social groups were maintained in the IntelliCage, as the number of cages and assignments of animals to them remained the same.

The sequence of experiments was as follows (one day referring to a timespan of 24 h, experiments were switched during the light phase):

- 2 days of Free Adaptation: open doors and free access to water bottles in all corners;
- 1 day of Free Adaptation, doors open on visit: doors open when a mouse entered a corner, free access to water bottles in all corners;

- 2 days of Nosepoke Adaptation: doors stayed closed until a mouse entered the corner and executed one or more nosepokes on a door; only the corresponding door opened and granted access to water for 7 seconds or until the mouse withdrew from the corner;
- 1 day of Nosepoke Adaptation at 50 %: same procedure as for Nosepoke Adaptation, but doors only opened with a probability of 50 % for each trial to counteract a bias for a specific corner by making it unreliable;
- 1 day of Nosepoke Adaptation at 30 %: same procedure as for nosepoke adaptation, but doors only opened with a probability of 30 % for each trial;
- 2 days place learning: each mouse was assigned to one of the four corners in a balanced fashion, ensuring equal distribution; mice could only get access to water after executing a nosepoke in their assigned corner;
- 7 days serial reversal learning: place learning with a new assignment of the drinking corner for each mouse every 24 hours, following a pre-defined, seemingly random order to make the new corner unpredictable for the mouse;
- 1 day sucrose preference: doors were open with free access to water bottles in all corners; one of the two bottles in each corner (each on the same side) was filled with a 4% sucrose solution, the other bottle contained normal drinking water.

Activity and nocturnality were assessed throughout the first five days. Place and sucrose preference as well as nocturnality of activity were measured using the preference score (A-B)/(A+B), weighted for random expectation (for place preference: (0.75 * A - 0.25 * B) / (0.75 * A + 0.25 * B)), where A equals the number of correct trials (visits in the assigned corner with at least one nosepoke), the number of licks at a sucrose solution bottle or visits during nighttime and B equaling incorrect trials (visits with nosepoke in non-assigned corner), licks at a bottle containing plain water or daytime visits.

Sequential Probability Ratio Testing (SPRT) was used to calculate the learning criterion for the assessment of learning performance over the serial reversal learning phases and learning flexibility after the first reversal (Wald, 1945). The learning criterion is the number of trials - defined as visits with at least one nosepoke - needed to pass the upper bound, i.e. a predefined learning criterion. The SPRT upper bound was defined as random expectation plus 10 % (35 % for four corners), the lower bound was equal to random expectation (25 % for four corners). Significance levels were set to 5 % for both bounds. In case a mouse did not reach the learning criterion within the duration of a reversal phase (24 hours), the total number of trials was used for downstream analysis and plotting instead. Overall serial reversal learning performance was measured as the approximated area under the curve across all reversal phases.

After Intellicage experiments, animals were transferred back to standard (type IV) cages and stayed in their established groups.

5 Pre-pulse inhibition

In advance to the actual pre-pulse inhibition testing, mice were habituated to the enclosures, white noise in the background, and lighting inside the soundproof test boxes for 10 minutes on three consecutive days at ZT6. Testing was performed on day four at ZT2.

Startle responses were measured automatically via movement-induced vibration of the base plate of startle-response-enclosures (SR-LAB, San Diego Instruments, San Diego, USA). The background noise in the boxes was set to a constant level of 65 dbA. For short term habituation, the main 40 ms 115 dbA pulse was presented ten times before the actual test sequence. To test pre-pulse inhibition, a non-startling 20 ms pre-pulse of an intensity of 70, 75, or 80 dBA was

presented, which was followed by a pulse of 115 dBA occurring 100 ms after the start of the pre-pulse. Additionally, the main pulse was presented alone for reference (baseline). Each condition was repeated in ten trials. All trials were presented in pseudorandomized order with inter-trial intervals ranging from 8 to 22 seconds.

6 Tail Suspension Test

Lack of active avoidance of an aversive situation was measured in the tail suspension test at ZT2. Adhesive tape was used to attach mice to a bar located 30 cm above a flat surface for 6 minutes. Plastic tubes were put over the tail to prevent mice from climbing up their own tail. The time immobile was quantified by measuring the amount of time when no whole-body movement was recorded. Whole-body movement was defined as movement of the center of the body. Flailing with the front limbs was not counted as movement. Mice were video-recorded and whole-body movements were quantified automatically using ANY-maze. Illumination during the test was set to 1600 lux.

7 Fear Conditioning

Fear conditioning was conducted using commercial soundproof boxes from Ugo Basile (Siena, Italy), which contain an enclosure with a grid floor that can deliver foot shocks, a dim light, and a speaker. The walls of the enclosure are covered with black and white striped paper slips for visual context. Animals were tested on three consecutive days at ZT2.

On day 1, experiment mice were conditioned. Animals were put into the box for the first time. For the first two minutes, baseline freezing behavior in a novel environment was recorded; after two minutes, an auditory cue was presented. After another 20 seconds, the tone ended, and a 2 second foot shock (0.6 mA) was delivered. Then, after another 30 seconds break the tone was repeated for 20 seconds with another consecutive foot shock for 2 seconds.

On day 2, animals were put back into their respective enclosure for 2 minutes without further intervention, while freezing behavior was recorded to assess the animal’s contextual fear memory.

On day 3, animals were put into their box again, but this time the enclosure had been replaced by a clear plastic 25 cm diameter pipe and a plain grey floor to avoid contextual fear. The pipe and grey floor were cleaned using SDS water, but not ethanol, again to avoid evoking contextual fear. After 2 minutes habituation time, the auditory cue was played for 2 minutes to assess cued fear memory.

Finally, the enclosure with the grid floor without striped paper slips was used to assess the pain threshold for each animal, measuring the minimum current necessary to evoke a visible pain reaction. This was done to avoid a bias in fear conditioning, introduced by reduced pain sensitivity.

Throughout all trials, the animal’s behavior was recorded by a video camera inside each box. Freezing behavior was scored automatically via ANY-maze.

8 Supplementary references

Hurst, J. L., and West, R. S. (2010). Taming anxiety in laboratory mice. *Nat. Methods* 7, 825–826. doi:10.1038/nmeth.1500.

Wald, A. (1945). Sequential Tests of Statistical Hypotheses. *Ann. Math. Stat.* 16, 117–186. doi:10.1214/aoms/1177731118.
